# Improved scientific knowledge of methanogenesis and methanotrophy needed to slow climate change during the next 30 years

**DOI:** 10.1128/mbio.02059-23

**Published:** 2023-09-21

**Authors:** Eric A. Davidson, Jeremy D. Semrau, Nguyen K. Nguyen, On behalf of Steering Committee and participants of the ASM/AGU Colloquium: The Roles of Microbes in Mediating Methane Emissions

**Affiliations:** 1 Appalachian Laboratory, University of Maryland Center for Environmental Science, Frostburg, Maryland, USA; 2 Spark Climate Solutions, San Francisco, California, USA; 3 Department of Civil and Environmental Engineering, University of Michigan, Ann Arbor, Michigan, USA; 4 American Academy of Microbiology, American Society for Microbiology, Washington, USA

**Keywords:** enteric, greenhouse gases, landfills, manure, methane, rice, waste management

## Abstract

Owing to the high radiative forcing and short atmospheric residence time of methane, abatement of methane emissions offers a crucial opportunity for effective, rapid slowing of climate change. Here, we report on a colloquium jointly sponsored by the American Society for Microbiology and the American Geophysical Union, where 35 national and international experts from academia, the private sector, and government met to review understanding of the microbial processes of methanogenesis and methanotrophy. The colloquium addressed how advanced knowledge of the microbiology of methane production and consumption could inform waste management, including landfills and composts, and three areas of agricultural management: enteric emissions from ruminant livestock, manure management, and rice cultivation. Support for both basic and applied research in microbiology and its applications is urgently needed to accelerate the realization of the large potential for these near-term solutions to counteract climate change.

## EDITORIAL

Methane is a potent greenhouse gas, contributing to about one-fourth of current positive radiative forcing and already having caused about 0.5°C warming since the beginning of the industrial revolution (1). Fortunately, methane has a short (about 12 years) atmospheric residence time. Consequently, effective abatement of methane emissions can be a fast-acting and important lever to slow climate change during the next 20–30 years (1).

The American Society for Microbiology and the American Geophysical Union, two of the largest scientific societies in the world, jointly convened a colloquium on “The Role of Microbes in Mediating Methane Emissions” on 31 May to 1 June 2023 to explore opportunities to improve methane mitigation options through improved understanding of the microbial processes of methane production and consumption. A group of 35 national and international experts from academia, the private sector, and government met to review the understanding of the microbial processes of methanogenesis and methanotrophy in waste management, including landfills and composts, and in three areas of agricultural management: enteric emissions from ruminant livestock (e.g., cattle and sheep eructation), manure management, and rice cultivation. Presentations on the state of the science in each of these topics revealed that much is already known about the biology of methanogens and methanotrophs (2 – 5), but important knowledge gaps remain for translating the science into reliable and cost-effective approaches to abate methane emissions from waste and agricultural sectors (6). For example, while feed additives have shown some promise in reducing livestock emissions of methane, an improved understanding of the budgets of energy and hydrogen that control methane production within the rumen, which currently are not well understood, could lead to discovering more solutions. In contrast, effective tools and designs already exist for landfills to capture methane for use as fuel and to reduce emissions further with methanotrophs in landfill soil covers, but new contributions from social science and economics are also needed to promote broader adoption of these technologies and to divert most organic wastes from landfills to composting facilities (6).

The colloquium participants found good reasons for optimism that microbial science is on the verge of facilitating significantly improved abatement of methane emissions from agriculture and waste management systems, but more basic and applied research is still urgently needed to accelerate the realization of the large potential for near-term solutions to climate change from methane emission abatement. Looking forward, the colloquium aims to articulate insights in the form of a roadmap to benchmark progress and to facilitate coordination of the scientific community toward a meaningful goal of methane emission reduction. A full report of the colloquium will be published by ASM in the autumn of 2023. In parallel, the National Academy of Sciences is also collecting information and preparing a report on the removal of atmospheric methane (7). Meanwhile, the 2023 Farm Bill currently being debated in the U.S. Congress provides a particularly propitious opportunity to advance R&D for methane abatement in the agricultural sector, where funding constraints have limited progress to date. These R&D needs include improved technologies for measuring ruminant methane emissions, improved understanding of rumen hydrogen and energy budgets, identifying effective rice management and manure management strategies to reduce both methane and nitrous oxide emissions, identifying barriers to re-integrating manure management with crop production systems, and investigating effective strategies to divert food wastes from landfills to proper composting facilities for agriculture and horticulture. Additional support for basic and applied research is also urgently needed from NSF, DOE, USDA, EPA, and other agencies. To meet the ambitious goals of the Global Methane Pledge (8) and to achieve near-term slowing of climate change, the best microbial science will be needed, and the ASM-AGU colloquium of experts demonstrated that a cadre of talented and enthusiastic experts is well positioned to contribute to this solutions-based science.
